# The phosphatase of regenerating liver-3 (PRL-3) is important for IL-6-mediated survival of myeloma cells

**DOI:** 10.18632/oncotarget.8422

**Published:** 2016-03-27

**Authors:** Tobias S. Slørdahl, Pegah Abdollahi, Esten N. Vandsemb, Christoph Rampa, Kristine Misund, Katarzyna A. Baranowska, Marita Westhrin, Anders Waage, Torstein B. Rø, Magne Børset

**Affiliations:** ^1^ K. G. Jebsen Center for Myeloma Research, Trondheim, Norway; ^2^ Department of Cancer Research and Molecular Medicine, Norwegian University of Science and Technology, Trondheim, Norway; ^3^ Clinic of Medicine, St Olavs University Hospital, Trondheim, Norway; ^4^ Department of Hematology, St Olavs University Hospital, Trondheim, Norway; ^5^ Department of Pediatrics, St Olavs University Hospital, Trondheim, Norway; ^6^ Department of Immunology and Transfusion Medicine, St Olavs University Hospital, Trondheim, Norway

**Keywords:** multiple myeloma, PRL-3, PTP4A3, IL-6, STAT-3

## Abstract

Multiple myeloma (MM) is a neoplastic proliferation of bone marrow plasma cells. PRL-3 is a phosphatase induced by interleukin (IL)-6 and other growth factors in MM cells and promotes MM-cell migration. PRL-3 has also been identified as a marker gene for a subgroup of patients with MM. In this study we found that forced expression of PRL-3 in the MM cell line INA-6 led to increased survival of cells that were depleted of IL-6. It also caused redistribution of cells in cell cycle, with an increased number of cells in G2M-phase. Furthermore, forced PRL-3 expression significantly increased phosphorylation of Signal transducer and activator of transcription (STAT) 3 both in the presence and the absence of IL-6. Knockdown of PRL-3 with shRNA reduced survival in MM cell line INA-6. A pharmacological inhibitor of PRL-3 reduced survival in the MM cell lines INA-6, ANBL-6, IH-1, OH-2 and RPMI8226. The inhibitor also reduced survival in 9 of 9 consecutive samples of purified primary myeloma cells. Treatment with the inhibitor down-regulated the anti-apoptotic protein Mcl-1 and led to activation of the intrinsic apoptotic pathway. Inhibition of PRL-3 also reduced IL-6-induced phosphorylation of STAT3. In conclusion, our study shows that PRL-3 is an important mediator of growth factor signaling in MM cells and hence possibly a good target for treatment of MM.

## INTRODUCTION

Multiple myeloma (MM) is a neoplastic proliferation of bone marrow plasma cells. The malignant cells are primarily located to the bone marrow (BM). Even though novel therapies have improved patient survival in recent years, relapse is still considered inevitable. [1] The biological and clinical behavior of MM cells is determined by genetic aberrations and the BM microenvironment. [2] One of the challenges in myeloma therapy is this redundant stimulation of MM cells by multiple signals in the BM microenvironment.

One of the most important survival signals for MM cells in the BM is given by interleukin (IL)-6. Binding of IL-6 to its receptor activates MAPK- and JAK/STAT3 signaling pathways. [3, 4] IL-6 enhances MM cell proliferation, survival, drug resistance and migration [5], and IL-6 levels in MM patients are correlated with advanced disease and a poor prognosis. [6, 7] Despite the fact that it is repeatedly shown that IL-6 is important in MM cell survival both *in vitro* and *in vivo*, treatment blocking IL-6 signaling has so far not proven to be an efficient approach. [8] Blocking one external survival signal will probably not be sufficient to eradicate MM cells in their growth factor-rich microenvironment. Since Phosphatase of regenerating liver (PRL)-3 is upregulated in response to IL-6 and other growth factors in MM cells [9], we have hypothesized that PRL-3 could be a downstream intersection where signals from several external growth factors converge, and possibly be a better treatment target.

Reversible phosphorylation of proteins is an important switch in multiple cellular processes. PRL-3 is a dual specificity phosphatase with ability to dephosphorylate tyrosine and serine/threonine residues. PRL-3 is encoded by the gene *PTP4A3* and its expression in cancer cells was shown to be associated with the cells’ ability to metastasize and with a poor prognosis in multiple cancers, including breast, colon, gastric, ovarian and esophageal carcinomas. [10] PRL-3 is also expressed in hematological malignancies. [9, 11, 12] In MM, PRL-3 has been shown to play a role in migration [9], and in acute myeloid leukemia (AML) it leads to drug-resistance. [11] Gene expression profiling studies done by Broyl *et al.* [13] identified 3 novel subgroups of MM, where one of the clusters was characterized by upregulation of *PRL-3*. The knowledge about PRL-3′s functional role in cancer, and in multiple myeloma in particular, is still limited. In this study we wanted to see to what extent PRL-3 is necessary and sufficient for mediating the effects of IL-6 on MM cells.

## RESULTS

### Forced expression of PRL-3 increased survival of INA-6 cells

**Figure 1:**
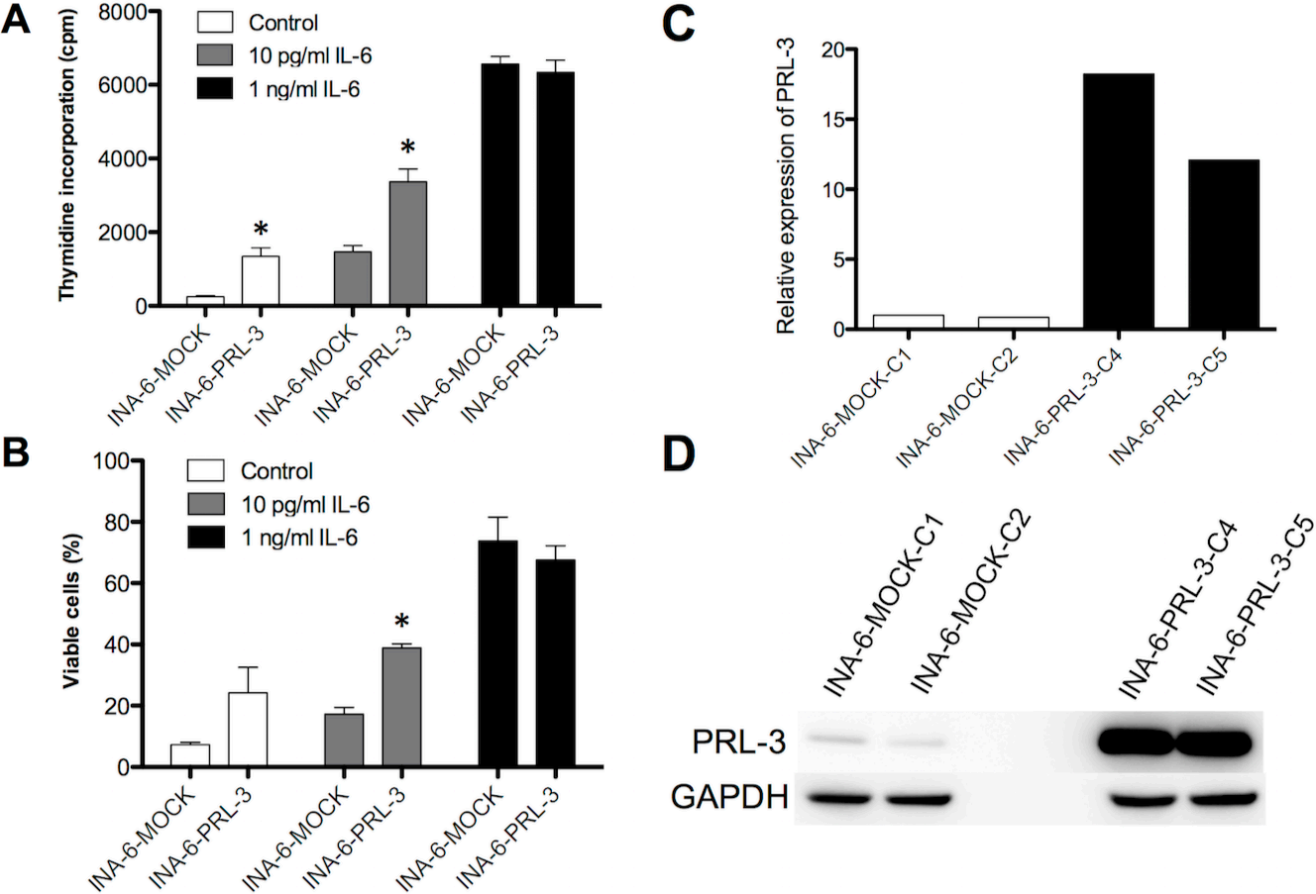
PRL-3 renders INA-6 cells less dependent on IL-6 Effect on ^3^H-thymidine incorporation (**A**) and survival as determined by annexin V-Alexa647 flow cytometry (**B**) of PRL-3 over-expression in human myeloma cell line INA-6, in the absence or the presence of suboptimal (10 pg/mL) or optimal (1 ng/mL) IL-6 concentrations. Error bars represent + 2 SD of quadruple (A) or duplicate (B) measurements. Figures show one representative experiment of three. Asterisk (*) denotes statistically significant difference from control (*P* < 0.05). PRL-3 overexpression in INA-6 cell line was confirmed by qRT-PCR (**C**) and by Western blotting (**D**).

The INA-6-WT cell line is dependent on IL-6 for its survival. IL-6 switches on PRL-3 expression in the cells. [9] By retroviral transduction, we made an INA-6 cell line variant overexpressing PRL-3 (INA-6-PRL-3) as confirmed in Figure 1C and 1D. Overexpression of PRL-3 in INA-6 cells gave a significant increase in thymidine incorporation in cells in the absence of or with a suboptimal concentration (10 pg/mL) of IL-6. In the presence of 1 ng/mL IL-6, which is a saturating concentration in this cell line, there was no significant difference in thymidine incorporation (Figure 1A). Whereas only 7% of INA-6-MOCK were viable after 4 days without IL-6, there were 24% viable cells in INA-6-PRL-3. Even though this difference was not significant, there was a clear tendency (*p* = 0,10) in 3 independent experiments. A suboptimal concentration of 10 pg/mL of IL-6 in INA-6-PRL-3 gave a significantly higher survival compared to INA-6-MOCK (Figure 1B). When cells were incubated with 1 ng/mL IL-6 there was no difference in survival (Figure 1B). To study whether the higher thymidine incorporation was due to increased proliferation or just reflected increased cell survival, we performed cell cycle analysis. We found a small but significant increase in cells in G2M phases in INA-6-PRL-3, and a small reduction in the percentage of cells in G1 and S phases (Table 1A and Supplementary Figure 1A). This finding indicates that PRL-3 causes a redistribution of cells in the cell cycle, but the lack of increase of cells in S-phase does not support a role of PRL-3 in proliferation. Using an inhibitor against PRL-3 (PRL-3 inhibitor I) we could not find significant changes in cell cycle distribution, but a tendency of increased number of cells in G1 and a reduction of cells in G2M (Table 1B and Supplementary Figure 1B). Taken together, these results indicate that PRL-3 overexpression makes the MM cell line INA-6 partially independent of IL-6 for survival, and that PRL-3 may influence cell cycle distribution.

**Table 1:** Distribution of cells in cell cycle with PRL-3 overexpression and inhibition

A						
	INA-6-MOCK No IL-6	INA-6-PRL-3 No IL-6	INA-6-MOCK 0,01 ng/mL IL-6	INA-6-PRL-3 0,01 ng/mL IL-6	INA-6-MOCK 1 ng/mL IL-6	INA-6-PRL-3 1 ng/ml IL-6
G1 (%)	27	22*	25	21	21	21
S (%)	59	54*	59	55*	59	55
G2M (%)	14	24*	16	23*	20	24*

B				
	INA-6 Control	INA-6 10 μM PRL-3 inh	INA-6 20 μM PRL-3 inh	INA-6 40 μM PRL-3 inh
G1 (%)	21	22	25	24
S (%)	57	57	60	58
G2M (%)	22	21	16	18

Cell cycle analysis using PI uptake and flow cytometry. Percentage of cells in G1, S and G2M phases of cell cycle. (**A**) INA-6-MOCK and INA-6-PRL-3 cultivated for 24 hours in the absence of IL-6 or with 0,01 ng/mL or 1 ng/ml IL-6. (**B**) INA-6 wild type cultivated for 24 hours in the presence of 1 ng/mL IL-6 and different concentrations of PRL-3 inhibitor I.

* denotes statistically significant difference from control group, *p* < 0.05.

### PRL-3 inhibition reduced survival in human myeloma cell lines

**Figure 2:**
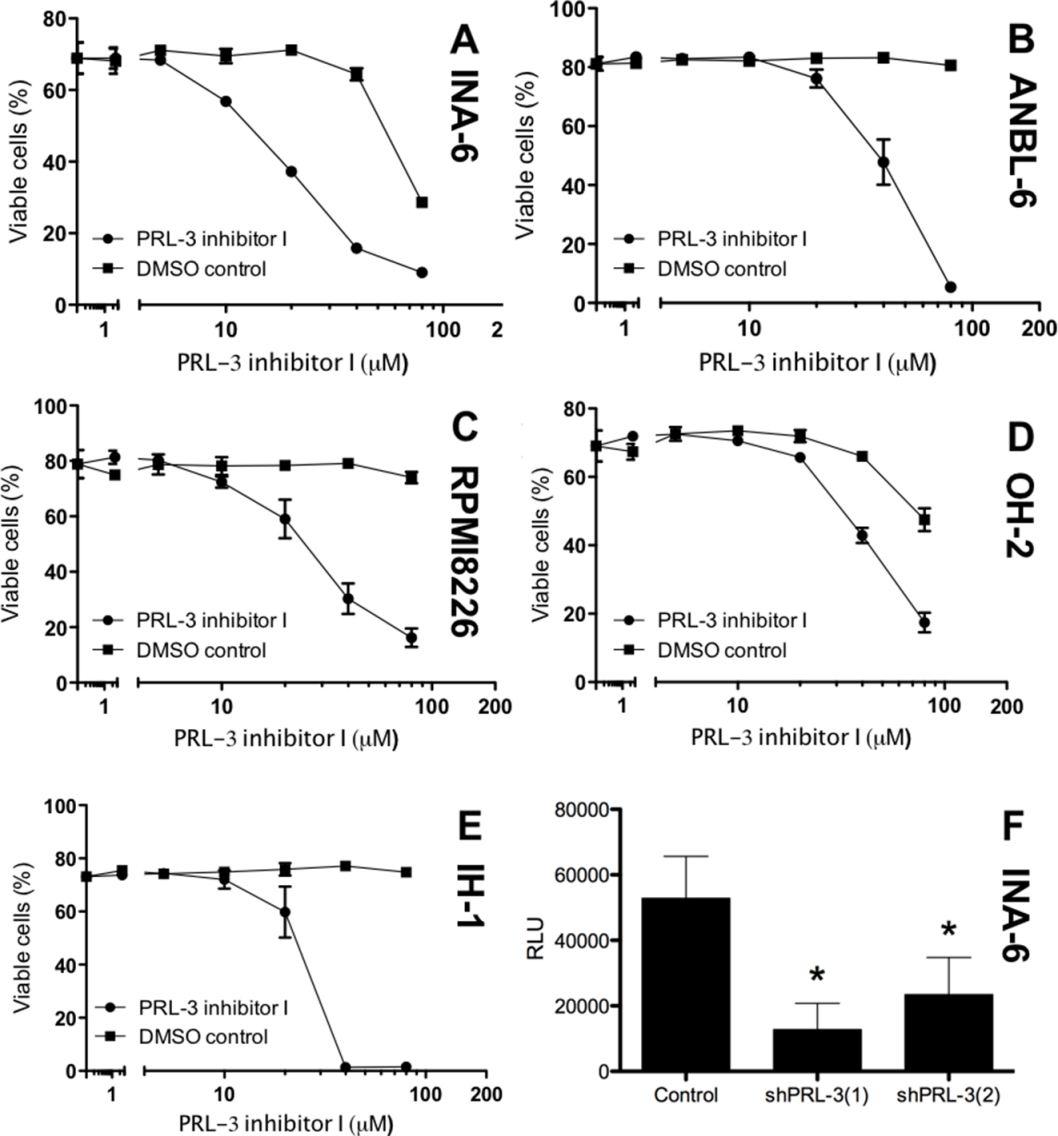
PRL-3 inhibition reduces survival in human myeloma cell lines PRL-3 inhibitor I inhibited survival of the 5 human myeloma cell lines INA-6-WT (**A**), ANBL-6 (**B**), RPMI8226 (**C**), OH-2 (**D**) and IH-1 (**E**). Cells grown for 3-4 days in 10% FBS or 0,1% BSA (**F**) in RPMI with 1 ng/mL IL-6 (INA-6-WT and ANBL-6), 10% FBS in RPMI (RPMI8226) or 2% HS in RPMI with 1 ng/mL IL-6 (IH-1 and OH-2). Cell viability was measured with annexin-V binding and PI uptake (A–E) or CellTiterGlo Luminescent Cell Viability Assay (F) Relative luciferase units (RLU) reflect the amount of ATP detected in each well. ShPRL-3(1) and (2) refer to knock down with two different shRNAs against *PRL3*. Error bars represent +/− 2 SD of triplicate (A–E) or quintuple (F) measurements. Figure showing one representative experiment of three. *Asterisks* in (F) indicates *P* ≤ 0.01.

We treated 5 HMCLs with increasing concentrations of PRL-3 inhibitor I. As seen in Figure 2A–2E, there was a dose-dependent reduction in survival in all cell lines tested. The IC_50_ value in cell lines INA-6-WT, ANBL-6, RPMI8226, OH-2 and IH-1 were 19 μM, 47 μM, 27 μM, 42 μM and 22 μM respectively. The PRL-3 inhibitor did not influence survival of BMSCs at concentrations applied in these experiments (data not shown), arguing against broad off-target effects of the inhibitor. We further did knock-down of endogenous PRL-3 with shRNA in the INA-6 cell line and found a clear reduction in viability in cells with reduced PRL-3 level.

### PRL-3 enhanced STAT3 signaling

**Figure 3:**
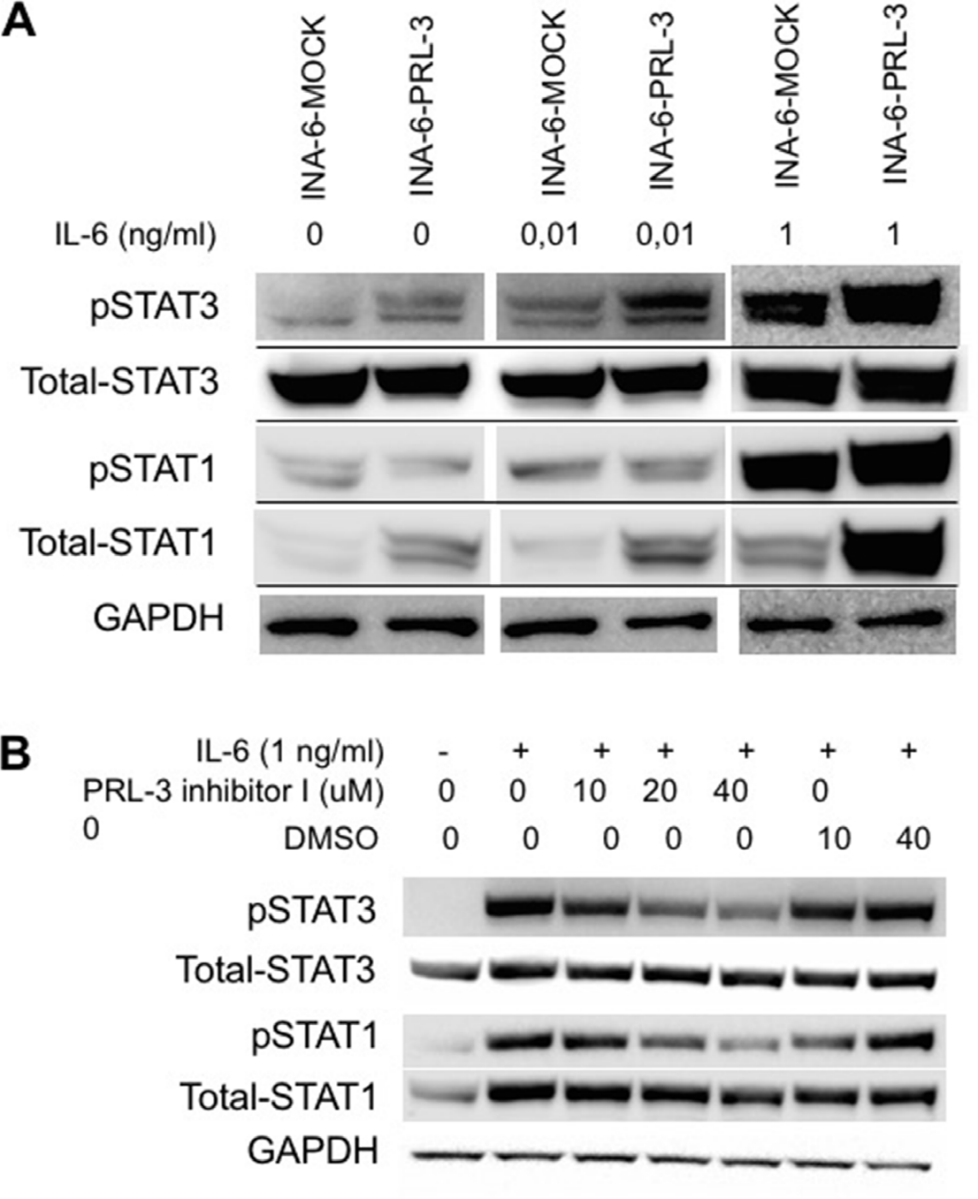
PRL-3 induces STAT-3 signaling (**A**) PRL-3 overexpression increased phosphorylation of STAT-3 (Y705) and total levels of STAT-1 in human myeloma cell line INA-6-WT. Cells washed 4 times and depleted of IL-6 for 3 hours before incubation without or with 10 pg/mL or 1 ng/mL IL-6 for 30 minutes. (**B**) INA-6-WT cells were starved for 3 hours, pre-incubated with inhibitor for 20 minutes before addition of IL-6. DMSO was used as control in inhibition experiments to exclude DMSO effects.

Next we wanted to investigate what impact PRL-3 had on signaling downstream from IL-6. Overexpression of PRL-3 increased phosphorylation of STAT3 (Y705) in the absence of IL-6, creating a constitutively active STAT3 signal. In the presence of 10 pg/mL or 1 ng/mL IL-6 there was a clear difference in STAT3 phosphorylation between INA-6-PRL-3 and INA-6-MOCK (Figure 3A). There was no difference in total STAT3 protein level. When using PRL-3 inhibitor I, there was a dose-dependent reduction in STAT3 phosphorylation upon IL-6 stimulation in INA-6-WT cells (Figure 3B). Collectively, these data indicate that PRL-3 influences the phosphorylation of STAT3, a signaling pathway important for myeloma cell survival. In addition, overexpression of PRL-3 led to increased expression of total STAT1 (Figure 3A), whereas the inhibitor decreased the level of total STAT1 (Figure 3B).

### PRL-3 inhibition reduced survival of primary MM cells, and co-cultivation of INA-6-WT cells with patient BMSCs increased PRL-3 expression

**Figure 4:**
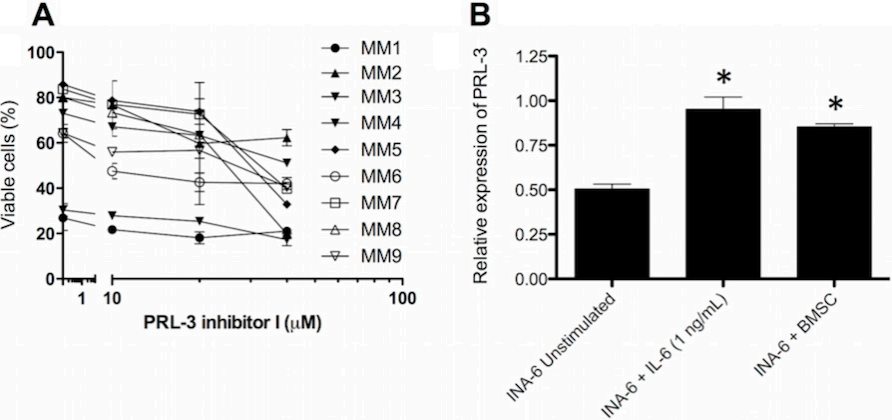
PRL-3 inhibition reduces survival in patient samples, and BMSC from myeloma patients induces PRL-3 expression (**A**) Primary myeloma cells were grown for 3 days in co-culture with BMSC pooled from 10 MM patients. The cells were cultivated in RPMI medium supplemented with 2% HS. Error bars represent +/− 2 SD of duplicate measurements. (**B**) PRL-3 mRNA levels in INA-6-WT cells. Co-cultivation experiments were performed as described in *materials and methods*. *PRL-3* mRNA levels in unstimulated INA-6-WT cells and INA-6-WT cells co-cultured with BMSC were normalized to that of INA-6-WT cells + IL-6. *GAPDH* expression was used as endogenous control. Error bars represent + 2 SD of duplicate measurements. Asterisk (*) denotes statistically significant difference from control group, *p* < 0.05.

We also evaluated the effect of PRL-3 inhibitor I on primary myeloma cells isolated from MM patients. In these experiments, cells were grown in co-culture with patient BMSCs to ensure higher viability of cells and an *in vitro* environment more similar to that of the patient bone marrow. [14] The PRL-3 inhibitor reduced survival in all of 9 primary cell samples tested (Figure 4A). To investigate whether the BM microenvironment could modify PRL-3 expression in myeloma cells, we co-cultured BMSCs from MM patients with INA-6-WT. This increased the concentration of PRL-3 mRNA to a level comparable to that in INA-6-WT cells stimulated with 1 ng/mL IL-6 and 1.6× to 1.8× higher than in INA-6-WT cells which were deprived of IL-6 for at least 5 hours (Figure 4B). This indicates that BMSC can contribute to myeloma cell survival by upregulating *PRL-3* expression in myeloma cells.

### PRL-3 inhibition reduced Mcl-1 expression and induced activation of the intrinsic apoptotic pathway

**Figure 5:**
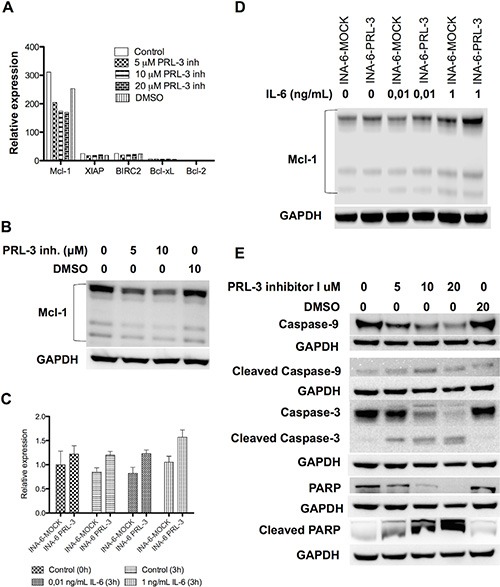
PRL-3 increases Mcl-1 expression, and PRL-3 inhibition reduces Mcl-1 expression and induces activation of the intrinsic apoptotic pathway (**A**) Expression of mRNA of the anti-apoptotic genes *Bcl-xL*, *Bcl-2*, *Mcl-1*, *XIAP* and *BIRC2* in INA-6-WT cells. Cells incubated for 24 hours with IL-6 (1 ng/mL) in 10% FBS in RPMI and increasing concentrations of PRL-3 inhibitor I. (**B**) Expression of Mcl-1 protein in human myeloma cell line INA-6-WT after incubation without or with 5 or 10 μM of PRL-3 inhibitor I for 24 hours. (**C**) Expression of Mcl-1 mRNA in INA-6-MOCK and INA-PRL-3, measured at start of experiment (0 h) and after 3 hours without or with 0,01 ng/mL or 1 ng/mL IL-6, respectively. (**D**) Expression of Mcl-1 protein in INA-6-MOCK and INA-6-PRL-3 after 3 hours without or with 0,01 ng/mL or 1 ng/mL IL-6, respectively. (**E**) Expression of Caspase-9, cleaved Caspase-9, Caspase-3, cleaved Caspase-3, PARP and cleaved PARP in human myeloma cell line INA-6-WT after treatment without or with 5, 10 or 20 μM of PRL-3 inhibitor I or DMSO for 24 hours. GAPDH was used as a loading control.

**Figure 6:**
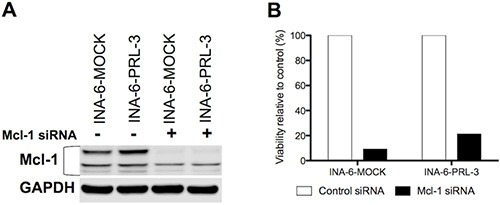
Mcl-1 is essential for MM cell survival (**A**) Expression of Mcl-1 protein in human myeloma cell lines INA-6-MOCK and INA-6-PRL-3 transfected with control- or Mcl-1-siRNA. (**B**) After transfection with siRNA, cells were grown for 48 hours in 10% FBS in RPMI with 1 ng/mL IL-6 before viability was determined by annexin V-Alexa647 flow cytometry. Percentage of viable Mcl-1 knockdown cells was calculated as relative to viable cells in control. Figure shows one representative experiment of three.

We treated INA-6-WT with increasing concentrations of PRL-3 inhibitor I for 24 hours in the presence of 1 ng/mL IL-6, and used qRT-PCR to screen for changes in expression of the anti-apoptotic genes *Bcl-xL*, *Bcl-2*, *Mcl-1*, *XIAP* and *BIRC2*. *Mcl-1* was the anti-apoptotic gene with highest expression in this cell line and the mRNA expression of *Mcl-1* was reduced in a dose-dependent manner after PRL-3 inhibition (Figure 5A). The INA-6-WT cells had almost no *Bcl-2* expression and low expression levels of the other anti-apoptotic genes tested (Figure 5A). 5 μM and 10 μM of PRL-3 inhibitor I gave a reduction in Mcl-1 protein expression (Figure 5B). To study if PRL-3 increases *Mcl-1* expression, we examined the expression in INA-6-PRL-3 and in INA-6-MOCK by qRT-PCR. The PRL-3-overexpressing cell line had a higher amount of *Mcl-1* both in the absence and the presence of IL-6. The same was seen for Mcl-1 protein (Figure 5D). We further studied how PRL-3 inhibition mediated apoptosis. Treatment for 24 hours with PRL-3 inhibitor I in increasing concentrations showed activation of Caspase-9, Caspase-3 and poly (ADP-ribose) polymerase (PARP), indicating activation of the intrinsic apoptotic pathway (Figure 5E). To study if Mcl-1 was essential for PRL-3-mediated myeloma cell survival we did knockdown experiments of Mcl-1 in INA-6-MOCK and INA-6-PRL-3 (Figure 6A–6B). Knockdown of Mcl-1 for 48 hours almost completely abrogated cell survival (Figure 6B).

## DISCUSSION

In the current study we have explored the significance of PRL-3 in multiple myeloma. We show that PRL-3 overexpression increased survival in the absence or with suboptimal concentrations of IL-6 in an IL-6-dependent cell line. Furthermore, we demonstrate that the use of an inhibitor against PRL-3 reduced myeloma cell survival in primary myeloma cells and HMCL. We identified new targets for PRL-3 in MM cells: PRL-3 overexpression increased STAT3 phosphorylation whereas PRL-3 inhibition reduced IL-6-induced STAT3 phosphorylation, down-regulated Mcl-1 and activated the intrinsic apoptotic pathway.

High PRL-3 expression is associated with cancer metastases and poor prognosis in various cancers. [10] In normal tissue, *PRL-3* mRNA expression has been found in skeletal muscle, pancreas and the heart, but also at lower levels in other organs, including hematopoietic cells. [15, 16] PRL-3 protein on the other hand, has not been detected in mature human tissues. [15]

To examine the role of forced PRL-3 expression in cells depleted of IL-6, we transduced INA-6 cells with retrovirus to stably overexpress PRL-3. Our results show that PRL-3 increased survival in the absence of IL-6 or at non-saturating concentrations of IL-6. At saturating concentrations of IL-6, PRL-3 overexpression did not increase survival, as expected since IL-6 switches on PRL-3. We also found changes in cell distribution with a significantly increased number of cells in G2M and a reduction of cells in S- and G1-phases in cells overexpressing PRL-3. We found a non-significant increase of cells in G1 in cells subjected to PRL-3 inhibition. This is in accordance with Fagerli *et al.* [9], which failed to show significant effect of PRL-3 knockdown by siRNA on proliferation or cell cycle distribution. Our data though indicate that high levels of PRL-3 change the distribution of cells in cell cycle. Previous data on PRL-3′s effect on proliferation of cancer cells are conflicting [17–19], possibly reflecting cell-type-dependent differences.

To further study how PRL-3 influenced survival in this IL-6-dependent cell line, we examined signaling downstream from IL-6. The IL-6-responsive transcription factor STAT3 was activated by PRL-3 expression, even in the absence of IL-6. Consistently, we found a dose-dependent reduction in STAT3 phosphorylation with the use of PRL-3 inhibitor I. Our data therefore support that PRL-3 expression can lead to a constitutively active STAT3 signal, thus rendering the IL-6-dependent MM cells less dependent on IL-6 for survival. A high level of STAT3 activation is found in the BM mononuclear cells of MM patients compared to normal controls [3]. This supports the role of constitutively active STAT3 in MM pathogenesis. Constitutive activation of STAT3 signaling mediates resistance to apoptosis in the MM cell line U266. [3] Brocke-Heidrich *et al.* [20] found that the IL-6-STAT3 signaling pathway only protected the cells from apoptosis and did not directly stimulate proliferation. This is supported by our results and the results of Fagerli *et al.* [9] showing that PRL-3, through STAT3 signaling, most likely influences survival but not proliferation. Others have also found an association between PRL-3 and STAT3 activation in both hematological [11] and non-hematological [21–22] cancers. *PRL-3* is also one of the genes most upregulated by IL-6 in the MM cell lines IH-1 and OH-2. [9] This up-regulation is most likely due to increased activation of the JAK/STAT3 signaling pathway downstream of IL-6R. Our data and the data of others therefore support that STAT3 signaling turns on PRL-3 expression and that PRL-3 increases STAT3 phosphorylation in a positive feedback loop. [9, 12] In addition, we observed that PRL-3 overexpression increased the expression of total STAT1 whereas PRL-3 inhibition decreased it. STAT1 and STAT3 are commonly considered to have opposing effects, where STAT1 have tumor suppressor properties and STAT3 may act as an oncogene. [23] The increased STAT1 expression in PRL-3 overexpressing cells could thus be a negative feedback mechanism.

We further demonstrate that inhibition of PRL-3 induced apoptosis and reduced survival in 5 MM cell lines. The inhibitor also reduced cell survival in patient samples, grown in co-culture with BMSCs from myeloma patients. As an alternative method we knocked down PRL-3 with shRNA in the INA-6 cell line. Similarly to the inhibitor, this caused a reduction in cell viability. The inhibitor did not affect the survival of BMSCs, indicating a myeloma-cell-specific effect in the bone marrow. The cells’ sensitivity to the inhibitor did not seem to be associated with their amount of PRL-3 protein. [9] This is consistent with the findings done on gastric cancer cell lines with use of the same PRL-3 inhibitor. [24] We found that the reduced survival upon PRL-3 inhibition was linked to a down-regulation of the anti-apoptotic protein Mcl-1. [25] Several studies show that Mcl-1 is an essential survival protein in MM cells, [26–28] and may therefore represent a significant barrier to the effectiveness of chemotherapeutic agents. It is shown that inhibition of Mcl-1 results in apoptosis and sensitization to chemotherapeutic drugs in various tumor cells, including multiple myeloma. [26, 29] In our study PRL-3 increased Mcl-1 expression and might be an explanation for the increased survival seen in PRL-3-overexpressing cells in the absence of IL-6. PRL-3′s influence on Mcl-1 could be due to the effects of PRL-3 on the STAT3 signaling pathway. It is previously shown that IL-6 up-regulates Mcl-1 in myeloma cells, [20, 30], which is abolished with an inhibitor of the JAK/STAT pathway. [30] Reduced expression of Mcl-1 will presumably facilitate homo- and heterodimerization of Bak and Bax and thus lead to mitochondrial outer membrane permeabilization, cytochrome c release, formation of the apoptosome and result in cleavage and activation of caspase 9. We here show that in cells treated with PRL-3 inhibitor I there was an increase in cleavage of caspase 9, caspase 3 and PARP, proving activation of the intrinsic apoptotic pathway by this inhibitor. Knockdown of Mcl-1 in both wild type and PRL-3-overexpressing myeloma cells confirmed that Mcl-1 is an essential survival factor in these cells.

In conclusion our data demonstrate a role of PRL-3 in MM cell survival, mediated, at least in part, by stabilizing the level of Mcl-1. Our results showing reduced MM cell survival after PRL-3 inhibition, makes PRL-3 a potential target in MM treatment. The reported lack of PRL-3 protein expression in adult tissue strengthens the possibility that PRL-3 can be an interesting target in cancer therapy. [15] However, since some adult tissues express *PRL-3* mRNA and since there has been a lack of very good and sensitive antibodies against PRL-3, one should not rule out a possible effect of PRL-3 protein in these cell's normal functions. Further studies, including *in vivo* experiments, are needed to evaluate the role of inhibitors of PRL-3 in MM therapy.

## METHODS

### Cell culture and treatment

We used the human myeloma cell lines (HMCL) IH-1 [31], OH-2 [32], RPMI8226 (from ATCC, Rockville, MD, USA), INA-6 (gift from Dr. M. Gramazki, University of Erlangen-Nuremberg, Erlangen, Germany) and ANBL-6 (gift from Dr. D. Jelinek, Mayo Clinic, Rochester, MN, USA). Cell lines were grown in RPMI-1640 supplemented with 2 mmol/L l-glutamine and 40 μg/mL gentamicin (referred to as RPMI). INA-6 and ANBL-6 were grown with 10% and RPMI8226 with 15% heat-inactivated fetal calf serum (FCS). IH-1 and OH-2 were supplemented with 10% human serum (HS) from our hospital's blood bank. INA-6, ANBL-6, IH-1 and OH-2 are IL-6-dependent cell lines and were maintained in media containing 1 ng/mL IL-6. Cells were cultured at 37°C in a humidified atmosphere with 5% CO_2_. Growth media were replenished twice weekly. Cell lines used in our laboratory are routinely tested for mycoplasma and new stock batches of the cell lines are thawed approximately every 4 months.

### Primary cells and bone marrow stromal cells (BMSC)

Patient CD138^+^ myeloma cells were isolated from bone marrow specimens using RoboSep automated cell separator and Human CD138 Positive Selection Kit (StemCell Technologies, Grenoble, France). Of separated cells > 90% were myeloma cells. Preparation of BMSC is described in detail by Misund *et al.* [14] Patient samples were obtained from the Norwegian Myeloma Biobank. All patients had given informed consent. The study was approved by the Regional Ethics Committee (approval #2011/2029).

### Antibodies, cytokines and other reagents

IL-6 was from Biosource (Camarillo, CA, USA). Antibodies against phosphorylated STAT3 (#9131), total STAT3 (#9132), phosphorylated STAT1 (#9167), total STAT1 (#14994), PARP (#9542), cleaved-PARP (#5625), Caspase-3 (#9662), cleaved-Caspase-3 (#9664), Caspase-9 (#9502) and cleaved-Caspase-9 (#9501) were from Cell Signaling Technology (Beverly, MA, USA). Antibody against Myeloid cell leukemia-1 (Mcl-1) (#819) and anti-PRL-3 (#318) were from Santa Cruz Biotechnology (Santa Cruz, CA, USA). Antibody against GAPDH was from Abcam (Cambridge, United Kingdom). In experiments, all compounds were diluted to final concentrations in RPMI. Cells were washed in Hanks’ balanced salt solution (HBSS) (Sigma-Aldrich, St. Louis, MO, USA). PRL-3 inhibitor I (5-[[5-Bromo-2-[(2-bromophenyl)methoxy]phenyl]methylene]-2-thioxo-4-thiazolidinone) was from Sigma-Aldrich (St. Louis, MO, USA). Gateway^®^ LR Clonase^®^ II Enzyme mix was from Invitrogen (Carslbad, CA, USA).

### Retroviral transduction for PRL-3 overexpression

To establish INA-6 cells expressing human *PTP4A3* (INA-6-PRL-3) and a control cell line (INA-6-MOCK), INA-6-WT cells were transduced with retrovirus that were made by transfecting Phoenix packaging cells with either pBMN-ires-GFP (control plasmid) or pBMN-PTP4A3-ires-GFP (plasmid containing the human PTP4A3 ORF cDNA). The pBMN-PTP4A3-ires-GFP was made by performing an LR recombination reaction between the ORF *PTP4A3* cDNA clone: ORFEXPRESS Gateway PLUS shuttle clone (GC-Z7908; GeneCopoeia, Rockville, USA) and the pBMN-CassetteA-IRES-GFP (made by blunt-end ligation of gateway cassetteA into multiple cloning site (MCS) of pBMN-ires-GFP) (Addgene plasmid 1736; Garry Nolan Lab). Cells were cloned by limiting dilution to yield individual clones, which were first checked for GFP expression, followed by analysis of *PTP4A3* mRNA and PRL-3 protein levels. All experiments with PRL-3-overexpressing cells were done with two independent sets of stably overexpressing cell lines, to reduce the possibility of differences because of peculiarities of the particular cell isolates.

### Thymidine incorporation assay

INA-6 cells overexpressing PRL-3 and vector-control cells were incubated in the absence of IL-6 or with 10 pg/mL or 1 ng/mL IL-6 and a thymidine incorporation assay was performed as described previously. [33].

### Apoptosis assay

Viability were evaluated by flow cytometry using annexin-V binding and PI uptake (APOPTEST-FITC kit; Nexins Research, Kattendijke, the Netherlands). Cells were washed 4 times in HBSS. 1,0 – 2,0 × 10^5^ (ANBL-6, IH-1, OH-2) or 0,5 – 1,0 × 10^5^ (INA-6, RPMI8226) cells were seeded in 24-well culture plates in 1 mL RPMI media containing 10% FCS with 1 ng/mL IL-6 (INA-6, ANBL-6), 10% FCS (RPMI 8226) or 2% HS with 1 ng/mL IL-6 (IH-1, OH-2). Cells were treated with PRL-3 inhibitor concentrations as indicated. DMSO controls were included since the inhibitor was dissolved in DMSO. In experiments with forced PRL-3 expression, 10^5^ of INA-6-PRL-3 or INA-6-MOCK were seeded in 1 mL RPMI media with IL-6 concentrations as indicated. After 2–4 days, cells were harvested and washed in phosphate-buffered saline (PBS), resuspended in 300 μL binding buffer, and incubated for 1 hour in the dark with 0,25 μL annexin V-flourescein isothiocyanate (FITC) or with 0,25 μl annexin V-Alexa647 (in cells transduced with GFP and CFP). In non-transduced cells 2 μL propidium iodide (PI) subsequently was added for 5 minutes. Flow cytometry was used to classify cells as annexin V-FITC (or Alexa647)- and/or PI-negative or –positive using BD LSRII Flow Cytometer (BD Biosciences, Franklin Lakes, NJ, USA). In experiments with shRNA knockdown of PRL-3 we measured viability using CellTiterGlo Luminescent (CTG) Cell Viability Assay (Promega, Madison, WI, USA). We followed the instructions provided by the manufacturer.

### ScanR cell viability assay

ScanR Cell Viability experiments were performed as described by Misund *et al.* [14].

### Immunoblotting

Cells were treated as indicated. Immunoblotting method was performed as described previously [33].

### Cell cycle analysis

INA-6 cells overexpressing PRL-3 and vector-control cells were washed 4 times in HBSS and seeded in 6-well cell culture plate (2,5–10^5^ cells/well) in 6 mL RPMI with 10% FCS. Cells were incubated in the absence of IL-6 or with 0,01 ng/mL or 1 ng/mL IL-6. In PRL-3 inhibition experiments INA-6-WT cells were incubated with PRL-3 inhibitor I in concentrations as indicated. After 24 hours cells were harvested and centrifuged, supernatants were removed and cells washed with PBS. Cells were then resuspended in 1 mL of ice cold 100% methanol. After removal of methanol, cells were washed once in PBS and incubated with 100 μg/mL RNAase (Ribonuclease A, Amresco Inc, Solon, OH, USA) for 30 minutes at 37°C. Cells where then incubated for 30 minutes at 37°C with 25 μg/mL PI in PBS. Cells were analyzed using a BD LSRII Flow Cytometer. Cell cycle analysis was performed in FlowJo 7.6 (TreeStar Inc, Ashland, OR, USA), after excluding aggregates and debris, using the Watson model.

### RNA isolation, cDNA synthesis and real-time PCR

Total RNA from INA-6-WT cell line was isolated with RNeasy Mini Kit (Qiagen, Crawley, United Kingdom). For samples of less than 5 × 10^5^ cells total RNA was isolated with RNAqueous^®^-Micro Kit (Life Technologies, Carlsbad, CA, USA). For cDNA synthesis, 1.0 μg total RNA was reverse-transcribed using the High Capacity RNA-to-CDNA kit (Life Technologies, Carlsbad, CA, USA), applying oligo(dT) primers. PTP4A3 (Hs00754750_m1), MCL-1 (Hs01050896_m1), BCL2L1 (Hs0236329_m1), BCL2 (Hs00608023_m1), XIAP (Hs00745222_s1), BIRC2 (Hs01112284_m1) and ENG (Hs00923996_m1) TaqMan^®^ primers were used to detect gene expression (Life Technologies, Carlsbad, CA, USA). The comparative DDCT-method was used for quantification using GAPDH (Hs99999905_m1) as endogenous reference.

### Measurement of PRL-3 mRNA in co-cultures

For the measurement of PRL-3 mRNA in cells maintained in co-cultures [34], BMSC were seeded at a concentration of 3 × 10^4^ cells per well (0.5 mL) into 24 well plastic plates and allowed to adhere for 24 h at 37°C in a humidified atmosphere containing 5% CO_2_. Then, 4 × 10^4^ INA-6-WT cells (0.1 mL) were added. INA-6-WT cells (4 × 10^4^ cells per 0.6 mL) cultured in absence of BMSC were maintained in presence of 1 ng/mL IL-6. Cultures were maintained at 37°C in a humidified atmosphere containing 5% CO_2._ After 48 h, cells were resuspended and co-cultures separated by CD138 selection according to manufacturer's instructions (Human CD138 Selection Kit, StemCell Technologies, Grenoble, France). Unstimulated cells were washed 5× with PBS and maintained for 5 hours at 37°C in serum free media in a humidified atmosphere containing 5% CO_2_. Harvested and purified cells were used for total RNA isolation, subsequent cDNA synthesis and determination of PRL-3 mRNA levels in each cell fraction by real-time polymerase chain reaction (PCR).

### Gene knockdown by siRNA nucleofection and shRNA

For gene knockdown by siRNA, INA-6-PRL-3 and INA-6-MOCK cells were grown with low density prior to the experiment. 5 × 10^6^ cells were pelleted, supernatant was discarded and the cell pellet was resuspended in transfection buffer (Amaxa^®^ Cell Line Nucleofector^®^ Kit R, Lonza, Switzerland). Cells were then added to separate nucleofection kuvettes containing 1 μM Mcl-1 siRNA (Mcl-1(S-19), Santa Cruz Biotechnology) and control siRNA (Control siRNA-A, Santa Cruz Biotechnology) and transfected by a Nucleofector™ II device (Lonza) (program X-001). Gel electrophoresis and Western blotting confirmed Mcl-1 knockdown. For gene knockdown with shRNA, Human Embryonic Kidney 293 cells (Hek-293) packaging cells were transfected with either 2 different PLKO-shRNA against PRL-3 and PLKO (control plasmid) in combination with packaging and envelope plasmid for virus production. Afterwards, INA-6 cells were transduced with viruses produced by packaging cells. PLKO and shRANA-PLKO against PRL-3 were a kind gift from Dr. Jim Lambert (University of Colorado, USA).

## SUPPLEMENTARY MATERIALS FIGURES


