# Supplementary material for: The phosphatase of regenerating liver-3 (PRL-3) is important for IL-6-mediated survival of myeloma cells
Source: Oncotarget. 2016 Mar 27;7(19):27295–306. doi: 10.18632/oncotarget.8422 (PMC5053650; doi:10.18632/oncotarget.8422)
Supplement: Supplementary file 1 [file oncotarget-07-27295-s001.pdf]

The phosphatase of regenerating liver-3 (PRL-3) is important for IL-6-mediated survival of myeloma cells

Supplementary Materials

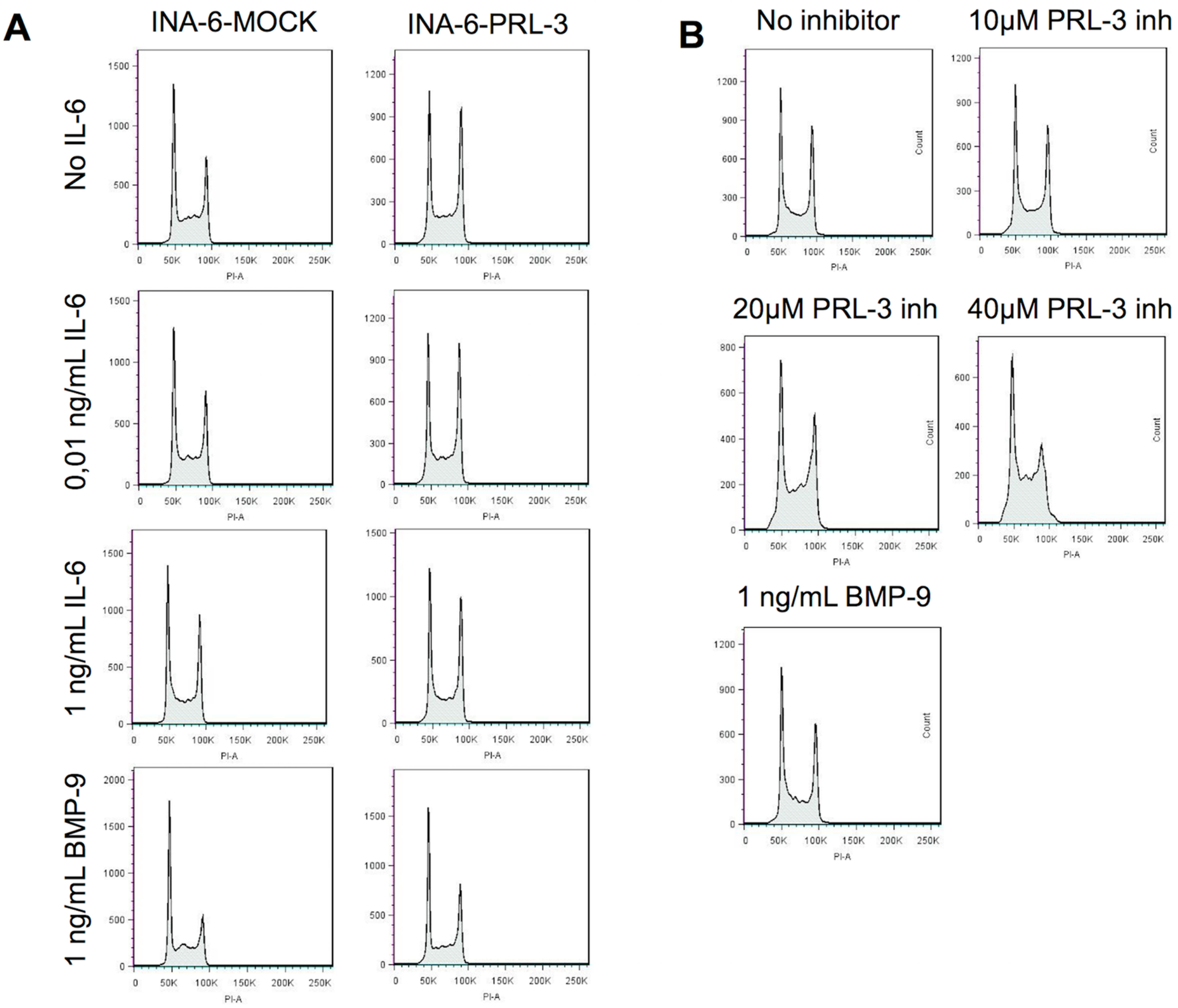

Supplementary Figure S1: Cell cycle analysis using PI uptake and flow cytometry. First peak are cells in G1-phase, second peak are cells in G2M-phases and the area in between are cells in Sphase. Percentages are calculated using the Watson model. (**A**) INA-6-MOCK and INA-6-PRL-3 cultivated for 24 hours in the absence of IL-6 or with 0,01 ng/mL, 1 ng/ml IL-6 or 1 ng/mL BMP-9 (**B**) INA-6 wild type cultivated for 24 hours in the presence of 1 ng/mL IL-6 and different concentrations of PRL-3 inhibitor I or 1 ng/mL BMP-9. BMP-9 is used as a positive control since it is known to cause cell cycle arrest in MM cells.

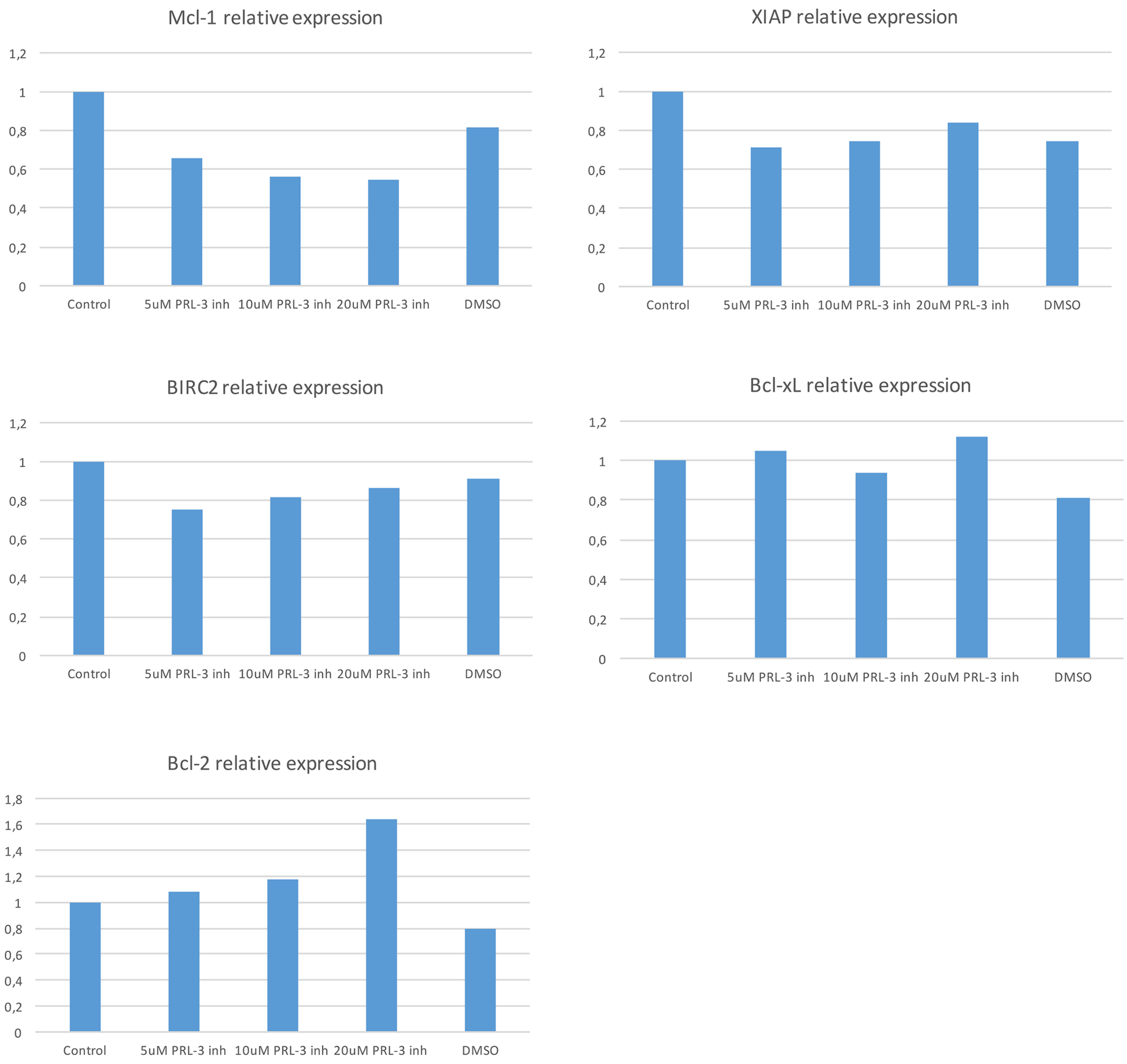

Supplementary Figure S2: Relative expression of genes shown in Figure 5A, normalized for each gene.
